# 3D- and 2D-QSAR models’ study and molecular docking of novel nitrogen-mustard compounds for osteosarcoma

**DOI:** 10.3389/fmolb.2023.1164349

**Published:** 2023-03-29

**Authors:** Wenkun Zhuo, Zheng Lian, Wenzhe Bai, Yanrong Chen, Huanling Xia

**Affiliations:** ^1^ Department of Orthopedics, The 960th Hospital of the Chinese People’s Liberation Army, Jinan, China; ^2^ Department of Orthopaedics, Affiliated Hospital of Shandong University of Traditional Chinese Medicine, Jinan, Shandong, China; ^3^ Department of Oncology, Jimo People’s Hospital, Qingdao, Shandong, China

**Keywords:** osteosarcoma, nitrogen-mustard drugs, 2D-QSAR, 3D-QSAR, drug design

## Abstract

**Background:** The dipeptide-alkylated nitrogen-mustard compound is a new kind of nitrogen-mustard derivative with a strong anti-tumor activity, which can be used as a potential anti-osteosarcoma chemotherapy drug.

**Objective:** 2D- and 3D-QSAR (structure–activity relationship quantification) models were established to predict the anti-tumor activity of dipeptide-alkylated nitrogen-mustard compounds.

**Method:** In this study, a linear model was established using a heuristic method (HM) and a non-linear model was established using the gene expression programming (GEP) algorithm, but there were more limitations in the 2D model, so a 3D-QSAR model was introduced and established through the CoMSIA method. Finally, a series of new dipeptide-alkylated nitrogen-mustard compounds were redesigned using the 3D-QSAR model; docking experiments were carried out on several compounds with the highest activity against tumors.

**Result:** The 2D- and 3D-QSAR models obtained in this experiment were satisfactory. A linear model with six descriptors was obtained in this experiment using the HM through CODESSA software, where the descriptor “Min electroph react index for a C atom” has the greatest effect on the compound activity; a reliable non-linear model was obtained using the GEP algorithm model (the best model was generated in the 89th generation cycle, with a correlation coefficient of 0.95 and 0.87 for the training and test set, respectively, and a mean error of 0.02 and 0.06, respectively). Finally, 200 new compounds were designed by combining the contour plots of the CoMSIA model with each other, together with the descriptors in the 2D-QSAR, among which compound I1.10 had a high anti-tumor and docking ability.

**Conclusion:** Through the model established in this study, the factors influencing the anti-tumor activity of dipeptide-alkylated nitrogen-thaliana compounds were revealed, providing direction and guidance for the further design of efficient chemotherapy drugs against osteosarcoma.

## 1 Introduction

Osteosarcoma originates from mesenchymal tissues and is characterized by osteoid matrixes produced by spindle tumor cells, which usually occurs in the epiphysis of the distal femur, proximal tibia, and proximal humerus, with pains, swelling, and local mass as the main symptoms. Pathological fractures are occasionally seen, meanwhile X-ray manifestations coexist with osteogenic and lytic lesions in the epiphysis of the affected diaphysis (Wittig et al., 2002). Before the 1970s, osteosarcoma was treated with amputation, but its 5-year survival rate was only 15%–20% (Marcove et al., 1970a; Marcove et al., 1970b). With the introduction of adjuvant chemotherapy in 1978, the disease-free survival rate of patients with primary osteosarcoma at the extremities has improved to 66%–75% (Bacci et al., 1998; Bacci et al., 2006). With further advances in chemotherapies, surgical techniques, and radiological staging, 90%–95% of patients with osteosarcoma can now receive limb salvage surgery and a functional reconstruction. The long-term survival rate and cure rate of local patients with tumors have reached 60%–80% (Wittig et al., 2002).

At present, first-line chemotherapy drugs against osteosarcoma mainly include methotrexate (MTX), Adriamycin (ADM), cisplatin (DDP), and ifosfamide (IFO) (Ferrari et al., 2014), which may play an important role in the treatment of osteosarcoma if combined in different ways. However, we should not ignore the side effects of these chemotherapy drugs, such as liver and kidney failure, severe gastrointestinal reactions, and bone marrow suppression (Becher et al., 1980; Allen, 1992; Tanihata et al., 2004). At the same time, the long-term use of a single chemotherapeutic agent may lead to drug resistance for tumor cells, which can ultimately be very harmful to patients with osteosarcoma (Lilienthal and Herold, 2020). Being disappointing in the past few decades, drug toxicity or drug resistance in chemotherapies for osteosarcoma recurrence and metastasis-related molecular mechanisms is not clear, e.g., the osteosarcoma chemotherapeutic progress has been stalled. Therefore, in order to further improve the cure rate of osteosarcoma, it is necessary to develop a less toxic and more effective chemotherapy drug against it.

The mechanism of nitrogen-mustard anti-tumor drugs is that they can form electron-deficient dimethylimine ions in the body and then covalently combine with electron-rich groups in biological macromolecules (such as DNA and RNA). Finally, nitrogen-mustard compounds destroy tumor-target DNA fragments, thus achieving the goal of eliminating tumor cells. (Liu et al., 2008). At the same time, nitrogen-mustard compounds have advantages, including simple synthesis and low cost, which have broad prospects in the clinical use of malignant tumor drugs

In recent years, a dipeptide-alkylated nitrogen-mustard compound has been found with a high anti-tumor activity, which brings new hope for the design of chemotherapy drugs against osteosarcoma (Chen et al., 2018; Singh et al., 2018).

In order to evaluate and design the activity of novel drugs more effectively and quickly, computer-aided experiment methods have been widely used. The structure–activity relationship quantification (QSAR) is an excellent experimental method for computer-aided drug design, through which the mathematical relationship between the chemical structure of a series of compounds and their pharmacological activity or other properties can be found in a quantitative way (Roy et al., 2015; Dearden, 2017).

In this study, we hope to establish a satisfactory prediction model of the anti-osteosarcoma activity of nitrogen-mustard compounds by using the QSAR method. This model can design chemotherapy drugs for osteosarcoma in the future.

## 2 Experiment

### 2.1 Dataset for analysis

In this experiment, all the 22 alkylated dipeptide nitrogen-mustard derivatives are from the study by Gullbo et al. (2003). The structure and bioactivity value of the 22 compounds are shown in Table 1.

**TABLE 1:** Structure and activity values of 22 compounds.

Structure	Substituent		IC_50_(µM)	NO
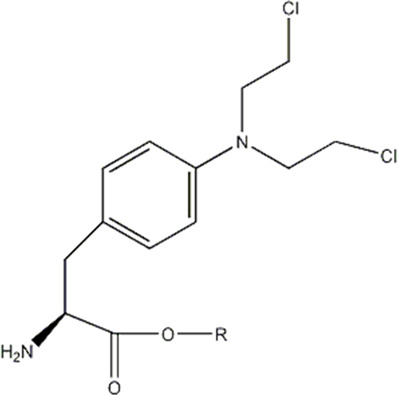	R= -H	/	1.7	I1
	R= -CH_2_CH_3_	/	6.1	I2
	R = -CH(CH_3_)_2_	/	5.3	I3*
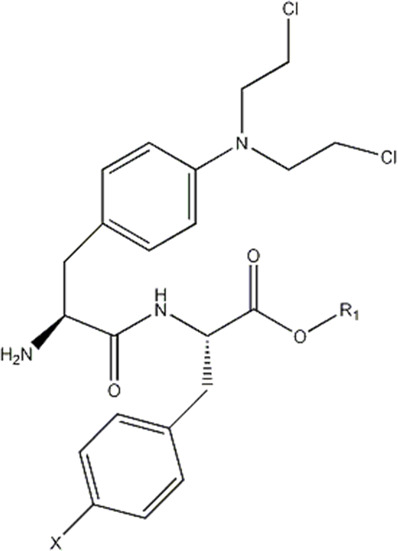	R_1_= -CH_2_CH_3_	X= -F	2.6	I4*
	R_1_= -CH_2_CH_3_	X= -H	1.9	I5
	R_1_= -CH_2_CH_3_	X= -OH	2.5	I6
	R_1_= -CH_2_CH_3_	X= -OCH_3_	1.8	I7
	R_1_= -CH_2_CH_3_	X= -NH_2_	2.7	I8
	R_1_= -CH_2_CH_3_	X= -NO_2_	2.0	I9
	R_1_= -CH_2_CH_3_	X= -N(CH_2_CH_2_Cl)_2_	2.5	I10
	R_1_= -CH(CH_3_)_2_	X= -F	1.9	I11
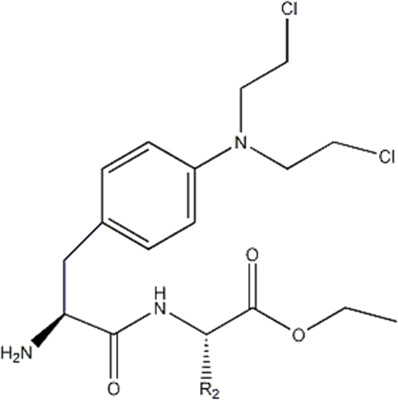	R_2_= -CH_2_-3-indoyl	/	4.6	I12
	R_2_= -CH_2_OH	/	19	I13
	R_2_= -CH_2_CH(CH_3_)_2_	/	4.2	I14*
	R_2_= -CH(CH_3_)_2_	/	12	I15
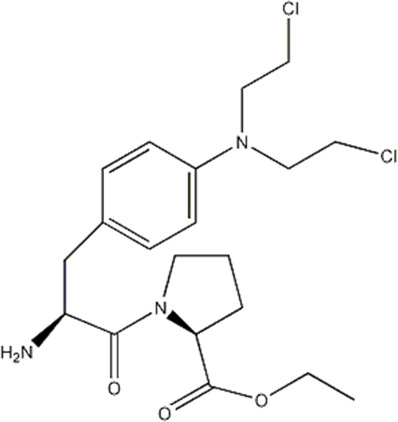	/	/	17	I16
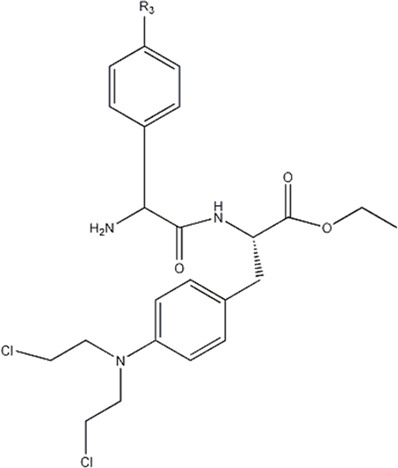	R_3_= -p-fluorobenzyl	/	5.7	I17
	R_3_= -CH(CH_3_)_2_	/	4.7	I18
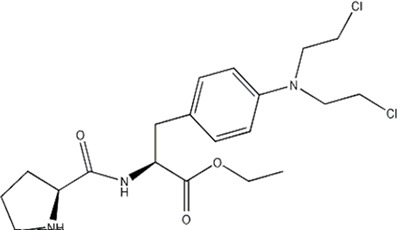	/	/	2.9	I19
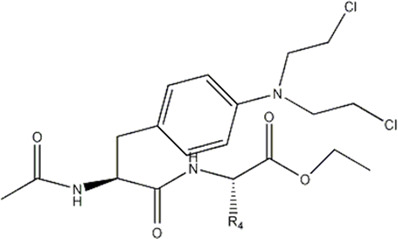	R_4_= -p-fluorobenzyl	/	3.0	I20*
	R_4_= -CH(CH_3_)_2_	/	11	I21
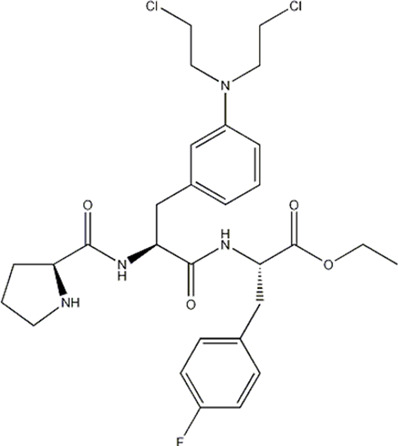	/	/	6.0	I22

**Note:** * represents the test set in the 2D-QSAR experiment, and the underline represents the test set in the 3D-QSAR experiment.

### 2.2 D-QSAR research

#### 2.2.1 Data processing and structure optimization

In order to obtain reliable experimental results, 22 compounds were grouped under random conditions using system time, 18 of which were in the training set for model construction, training, and optimization. The test set contained four compounds, which would be used to assess the predictive power of the model.

The key to building a good predictive QSAR model is to use and define molecular descriptors properly. So, the optimization of compounds is extremely important.

In this experiment, all compounds were constructed using ChemDraw software, which were then imported into HyperChem software. First, an MM + molecular mechanic field was used for rough optimization. In the second step, a precise optimization was performed using semi-empirical AM1 or PM3 methods using HyperChem, and the molecular structure was optimized using the Polak–Ribiere algorithm, until the root mean square gradient was 0.01 (Stewart, 1989; HyperChem, 1994). Finally, the results were imported into CODESSA software (Katritzky et al., 2001) to calculate five classes of molecular descriptors: constitutional, geometrical, topological, electrostatic, and quantum chemical.

#### 2.2.2 Linear model through a heuristic method (Cao and Lin, 2003)

Feature selection is used to reduce the number of descriptors and delete those that have less impact on the result. The remaining descriptors should represent the molecular structure and various properties as much as possible. The HM implemented in CODESSA software is used to calculate molecular descriptors and build linear models, and there is no software limitation on the size or speed of the dataset.

The detailed steps to establish a linear model through HM are as follows: selecting a parameter descriptor according to R^2^, F-test, t-test, and R^2^cv. After obtaining the two-parameter correlation coefficient with the best statistical characteristics, we added descriptors that are not used in the previous selection process. We repeat this step until the established correlation equation contains the most parameters. As a result, a linear model with six descriptors was developed through HM.

#### 2.2.3 Non-linear model through GEP

**FIGURE 1:**
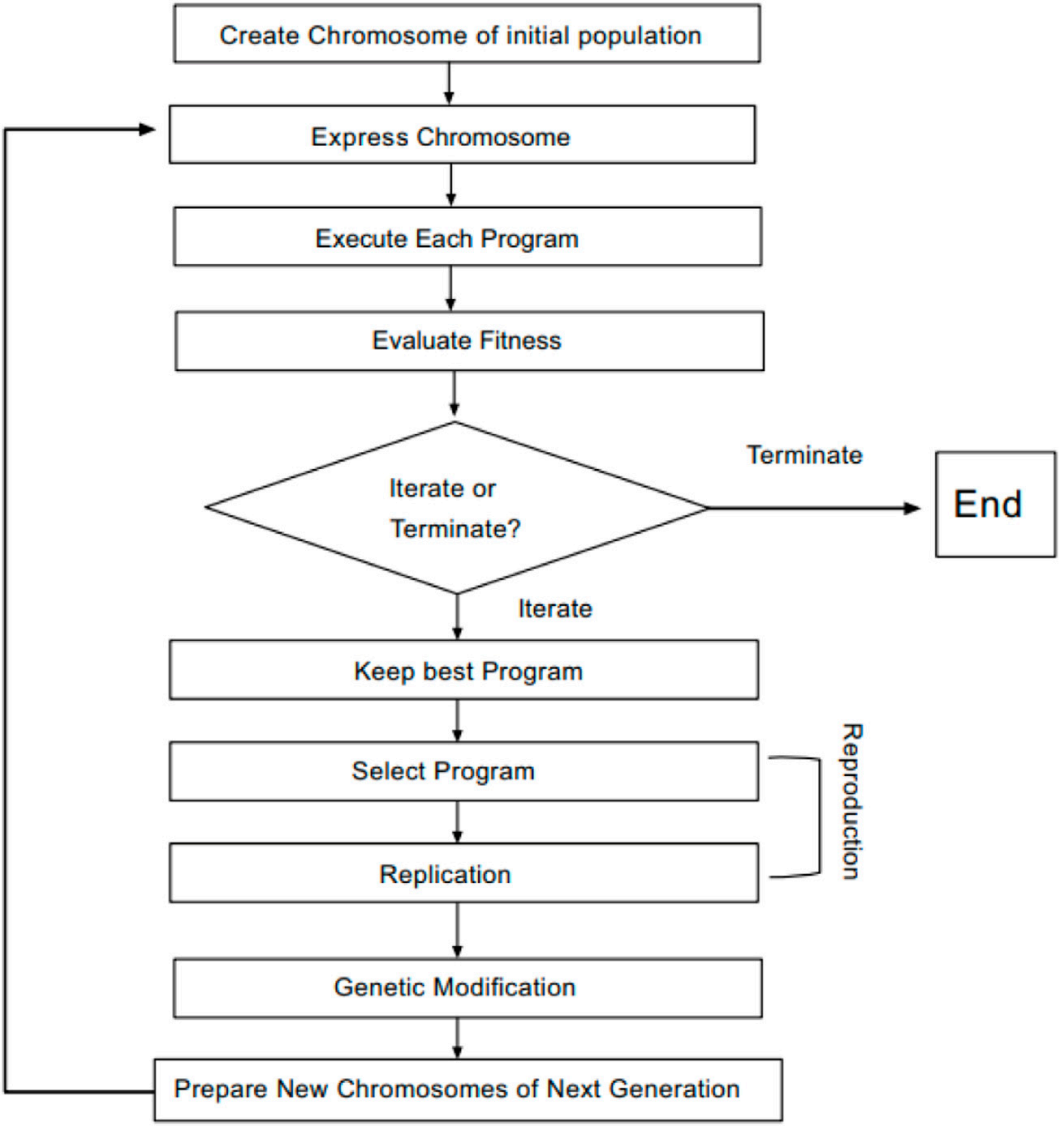
GEP flowchart.

The principles of gene expression programming (GEP) are as follows: GEP is developed from the genetic algorithm (GA) and genetic programming (GP) (Holland, 1992), in which some limitations of GA and GP are overcome (TeodorescuSherwood, 2008). Therefore, the efficiency of the GEP algorithm is much higher than that of the aforementioned two algorithms. GEP is considered to be an evolutionary algorithm based on Darwin’s theory of the survival of the fittest (Pham, 2012). Compared with genetic algorithms based on encoded numbers and GP based on an analytical tree, a candidate solution of GEP is linear chromosomes (Kaydani et al., 2014). A chromosome consists of more than one gene divided into two parts, a head and a tail. The header section can be selected from the end set and feature set, while the tail section can only be selected from the end set (TeodorescuSherwood, 2008). Finally, these genes are decoded into expression trees (ETs) (Gharagheizi et al., 2012) to obtain mathematical equations. The basic steps of the GEP algorithm are shown in Figure 1. First, a certain number of individual chromosomes are randomly generated to be expressed as the initial population. Next, the fitness of each individual is calculated based on a set of fitness samples. Then, if a solution of an ideal quality is found, or a certain number of iterations are run, the process can be terminated (TeodorescuSherwood, 2008). Otherwise, these individuals will be selected for genetic manipulation based on their fitness values. Finally, offspring with new characteristics is produced. We repeat the process until we obtain a good result.

In this study, we import the values of descriptors into automatic problem solver (APS) and integrate them with the GEP algorithm to obtain non-linear models. In order to obtain a good non-linear model, we select appropriate functions and evaluate their fitness through R^2^.

By comparing linear models with non-linear ones, it is found that non-linear models obtained through the GEP algorithm are more stable with better prediction ability. However, a 2D-QSAR model still cannot be used to accurately describe the relationship between molecular three-dimensional structures and their physiological activity, so it is necessary to continue 3D-QSAR experiments.

### 2.3 3D-QSAR research

#### 2.3.1 Data processing and structure optimization

Like the previous 2D-QSAR experiments, in 3D-QSAR experiments, the dataset also needs to be divided into a training set and a test set. The training set containing 18 compounds will be used to build the models, and the test set containing four compounds will be used to verify them. At the same time, in order to reduce the deviation caused by the dataset to the experimental results, −log (IC_50_) + 6 is used to convert the IC_50_ value in the subsequent experiments.

In the previous experiments, ChemDraw software was used to construct all the 22 compounds, while in 3D-QSAR experiments, they are put into SYBYL software for optimization and modeling. When processing data using SYBYL software, the Tripos force field and Powell’s gradient algorithm are used to minimize CoMSIA structure energy. Finally, the minimal structure is used as the initial conformation (Yu et al., 2015).

#### 2.3.2 Conformational sampling and alignment

**FIGURE 2:**
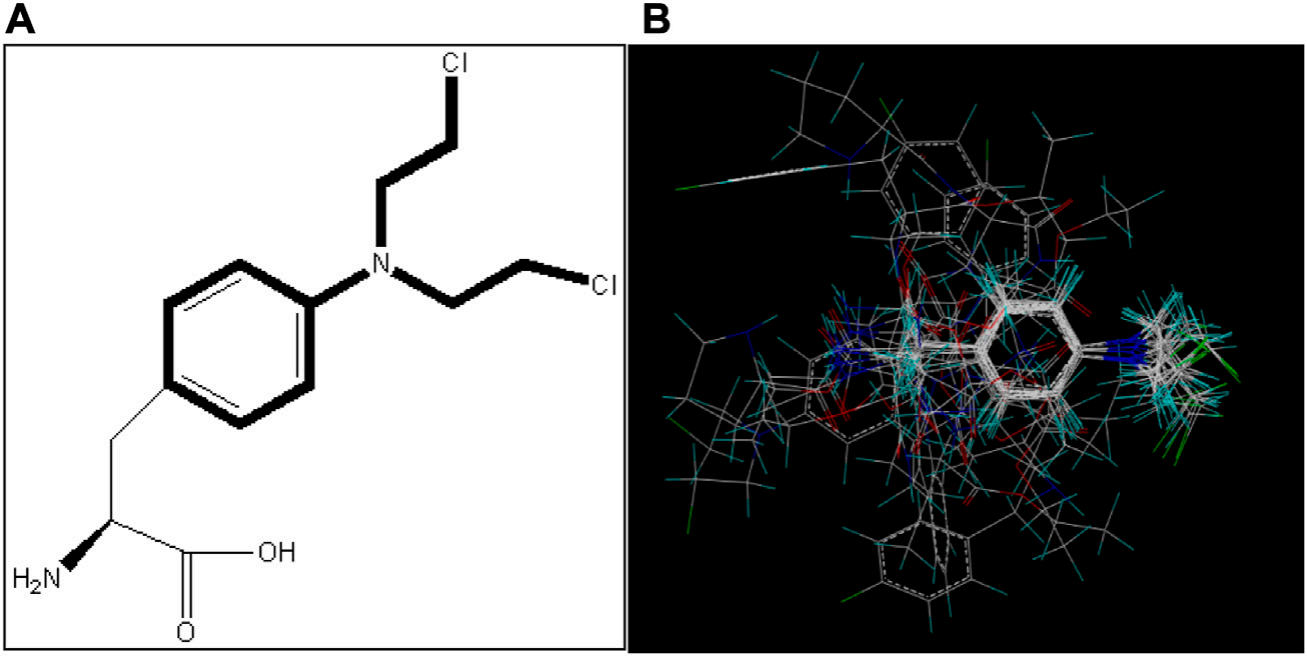
Alignment of all compounds in the dataset; compound I1 is used as a template. **(A)** Structure of compound I1 and the common substructure (shown in bold) for the alignment of all compounds. **(B)** Alignment of all the compounds.

In 3D-QSAR analyses, the structure comparison of compounds will directly affect the structure of subsequent tests; so, it is very important to select an appropriate comparison of compound structures (Yan et al., 2005; Patel et al., 2008; Yong et al., 2011). In this study, ligand alignment is used to superpose the structure of compounds, and the superposition patterns of all compounds can be seen in Figure 2. Because compound I1 has the highest IC_50_ value, all compounds are aligned with it in this method.

#### 2.3.3 CoMSIA study

CoMSIA is an excellent 3D-QSAR research tool (Yu et al., 2015). In the CoMSIA method, the Gaussian function related to distance is used to calculate various molecular fields, which can effectively avoid significant changes of potential energy and abnormal atomic positions at lattice points near the molecular surface. In addition, in CoMSIA, it is no longer necessary to define the cut-off value of energy. Compared with CoMFA, the correlation isosurface diagram of the contribution in different molecular fields of corresponding spaces of CoMSIA is significantly improved, which can be used to more intuitively explain the effect of different molecular fields on molecular activity (Li et al., 2012a). A CoMSIA study is carried out using the SYBYL software package, in which five molecular fields are used: spatial field (S), electrostatic field (E), hydrophobic field (H), hydrogen bond donor (D), and hydrogen bond acceptor (A). The CoMSIA method is calculated based on a 3D cubic lattice with a grid spacing of 2 Å and extending 4 Å units beyond the aligned molecules in all directions. A default value of 0.3 is used for the attenuation factor α (Yang et al., 2011a).

Partial least squares (PLS) analysis was used to correlate CoMSIA fields with −log (IC_50_) + 6 values to generate a statistically significant 3D-QSAR model, which was performed in two stages (Hadni and Elhallaoui, 2020). First, a leave-one-out cross-validation analysis was performed to determine the optimal group score (ONC) and cross-validation correlation coefficient (Q^2^). Then, the ONC was used in a non-cross-validation analysis to generate the final PLS regression model for CoMSIA. The non-cross validation results were evaluated based on several statistical parameters, including non-cross validation correlation coefficient (R^2^), estimated standard error (SEE), and F-value (Yan et al., 2020).

#### 2.3.4 Validation of the 3D-QSAR model

In order to prove the stability of the QSAR model, the 3D-QSAR model needs to be evaluated using internal or external validation methods (Yan et al., 2020). In this experiment, external validation was selected to verify the 3D-QSAR model. The verification formula is as follows:

$$R_{ext}^{2}=1-\frac{\sum_{i=1}^{ntest}{\left(y_{i}-{\tilde{y}}_{i}\right)}^{2}}{\sum_{i=1}^{ntest}{\left(y_{i}-{\tilde{y}}_{tr}\right)}^{2}}$$

In this formula, *ntest* refers to the number of compounds in the test set, ${\tilde{y}}_{tr}$ refers to the average value of compound activity in the training set, and $y_{i}$ and ${\tilde{y}}_{i}$ refer to the experimental value and predicted value of compound activity in the test set, respectively. Generally, with $R_{ext}^{2}$ > 0.5, the established model is considered robust with a good predictive ability in statistics (Yang et al., 2011b; Mouchlis et al., 2012).

## 3 Results

### 3.1 The results of the HM

**FIGURE 3:**
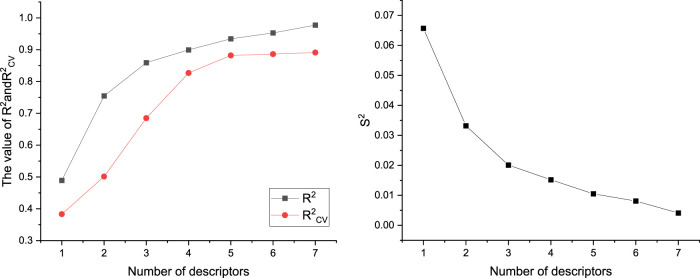
Effects of different number of descriptors on R^2^, R^2^_cv_, and S^2^.

A total of 526 molecular descriptors of 22 compounds were calculated using CODESSA software. In order to find the best linear model, the HM was used to construct linear models with 1–7 descriptors, respectively. The R^2^, R^2^_cv_, and S^2^ of these models are shown in Figure 3. The results showed that with the increase of the number of descriptors, R^2^ and R^2^_cv_ increased, while S^2^ decreased. After a comprehensive consideration, a model with six descriptors was selected as the best linear model to predict inhibitor activity (Table 2).

**TABLE 2:** Selected molecular descriptors and their physical-chemical meaning, coefficient, and t-test.

Symbol	Physical-chemical meaning	Coefficient	T-test
AVC	Avg valency of a C atom	8.2976e+01	8.8248
Min ERC	Min electroph react index for a C atom	−2.4177e+04	−8.6408
Min ECC	Min exchange for a C–C bond	2.9565e+00	6.2876
Min TCH	Min total interaction for a C-H bond	−1.9489e+00	−5.0704
NN	Number of N atoms	−1.7856e-01	−3.8961
TDM	Tot dipole of the molecule	5.1500e-02	2.5964

**FIGURE 4:**
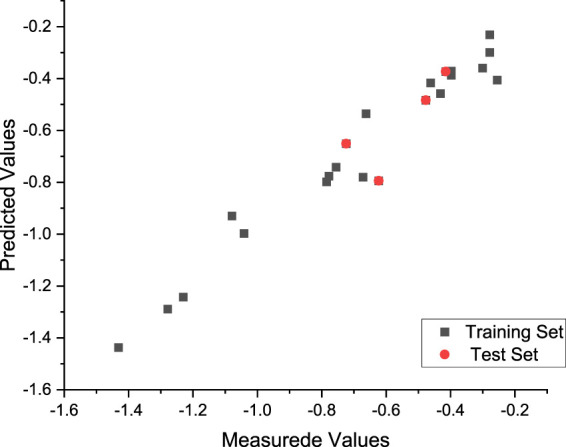
Plot of the measured and calculated −log (IC_50_) by HM.

In addition, to avoid the multicollinearity of molecular descriptors, correlation coefficients of those descriptors were calculated, as shown in Table 3. The results showed that the correlation coefficients of any two descriptors were less than 0.8, which meant that all descriptors were independent. Therefore, the linear model constructed in the experiment has strong statistical reliability (Figure 4).

**TABLE 3:** Correlation matrix of the six descriptors.

Name	A	B	C	D	E	F
1	1.0000	0.0303	0.0788	0.0340	0.1254	−0.5993
2	0.0303	1.0000	−0.1605	0.1743	−0.4819	−0.1196
3	0.0788	−0.1605	1.0000	−0.5146	0.3175	−0.1058
4	0.0340	0.1743	−0.5146	1.0000	−0.4512	0.2609
5	0.1254	−0.4819	0.3175	−0.4512	1.0000	−0.0609
6	−0.5993	−0.1196	−0.1058	0.2609	−0.0609	1.0000

**Note:** The letters A, B, C, D, E, and F represent AVC, Min ERC, Min ECC, Min TCH, NN, and TDM, respectively, and the numbers 1, 2, 3, 4, 5, and 6 represent AVC, MIN ERC, MIN ECC, MIN TCH, NN, and TDM, respectively.

The linear model equation is as follows:

$$-\log\;\left(IC_{50}\right)=-6.3254+8.2976*10^{1}*AVC-2.4177*10^{4}*\mathrm{Min}-ERC+2.9565*\mathrm{Min}-ECC-1.9489*\mathrm{Min}-TCH-1.7856*10^{-1}*NN+5.1500*10^{-2}*TDM.$$

According to the absolute value of the coefficients in the formula, it can be seen that the influence of descriptors on the anti-tumor activity of nitrogen-mustard compounds is as follows:

Min−ERC>AVC>Min−ECC>Min−TCH>NN>TDM.

### 3.2 The results of GEP

The dataset was randomly divided into a training set containing 18 compounds and a test set containing four compounds, and then, a non-linear model was built using software automatic problem solver (APS) to integrate the implementation of the GEP algorithm (Table 4).

**TABLE 4:** Operator symbols and parameters of the regression equation.

Parameter name	Representation	Value
Addition	+	1
Subtraction	-	1
Multiplication	*	1
Division	/	1
Natural logarithm	Ln	1
Sine	sin	1

**FIGURE 5:**
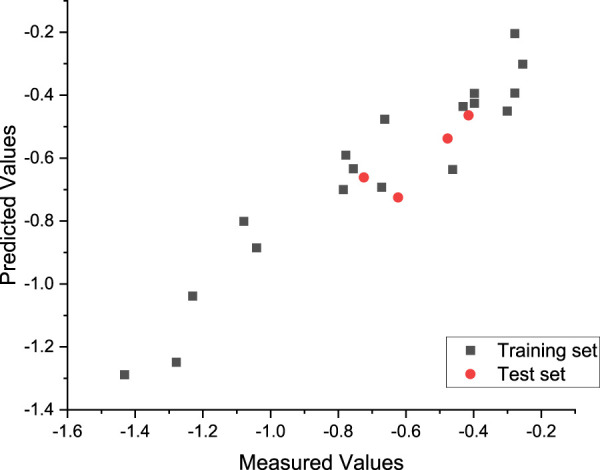
Plot of the measured and calculated −log (IC_50_) by GEP.

Finally, the best model was generated in the 89th generation cycle. The correlation coefficient of the training set and test set was 0.95 and 0.87, respectively, and the average error was 0.02 and 0.06, respectively (Figure 5).

Moreover, the non-linear model equation decoded by ETs was as follows:

$$\mathrm{Sin}\left(X_{0}\right)+\left(\mathrm{Sin}\left(X_{2}/X_{1}\right)+X_{4}/X_{5}\right)/X_{4}/X_{2}+\mathrm{Sin}\left(X_{3}*X_{3}*X_{1}\right)*\left(X_{1}/X_{3}X_{3}*X_{3}\right)+\mathrm{Sin}\left(\mathrm{Sin}\left(X_{0}\right)*X_{0}*X_{0}*X_{0}\right)+\mathrm{Sin}\left(\mathrm{Sin}\left(X_{2}\right)+\mathrm{Sin}\left(\mathrm{Sin}\left(X_{2}\right)*X_{0}\right)\right).$$

### 3.3 CoMSIA statistical results

The statistical results of the best CoMSIA model are shown in Table 5. Through the CoMSIA model, we derived a q^2^ of 0.532 with an optimum number of components, which was five. A high r^2^ of 0.997 with a low SEE of 0.016 and an F-value of 1601.378 were obtained.

**TABLE 5:** Statistical results of the optimal CoMSIA model.

Model	q^2^	ONC	r^2^	SEE	F
CoMSIA	0.532	5	0.997	0.016	1601.378

Name	S	E	H	D	A
Contribution (%)	5.4	27	22.7	26.3	18.5

### 3.4 CoMSIA model validation results

**FIGURE 6:**
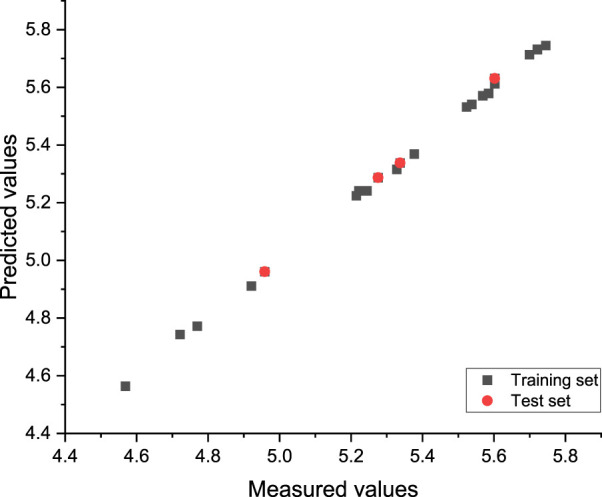
CoMSIA model-predicted activity values compared with the experimental values.

In this experiment, an external validation formula was used to verify the 3D-QSAR model constructed in the experiment. The value of R^2^_ext_ was 0.987, which was greater than 0.5, indicating that the established model had strong stability and good statistical prediction ability. Figure 6 shows a good relationship between the predicted values and the experimental values.

### 3.5 CoMSIA contour maps

The contour map of a CoMSIA model can clearly show the influence of drug groups on drug activity in different molecular force fields. Therefore, in the design and development of new drugs, more effective and excellent drugs can be designed according to the contour map (Li et al., 2012b; Mao et al., 2012).

**FIGURE 7:**
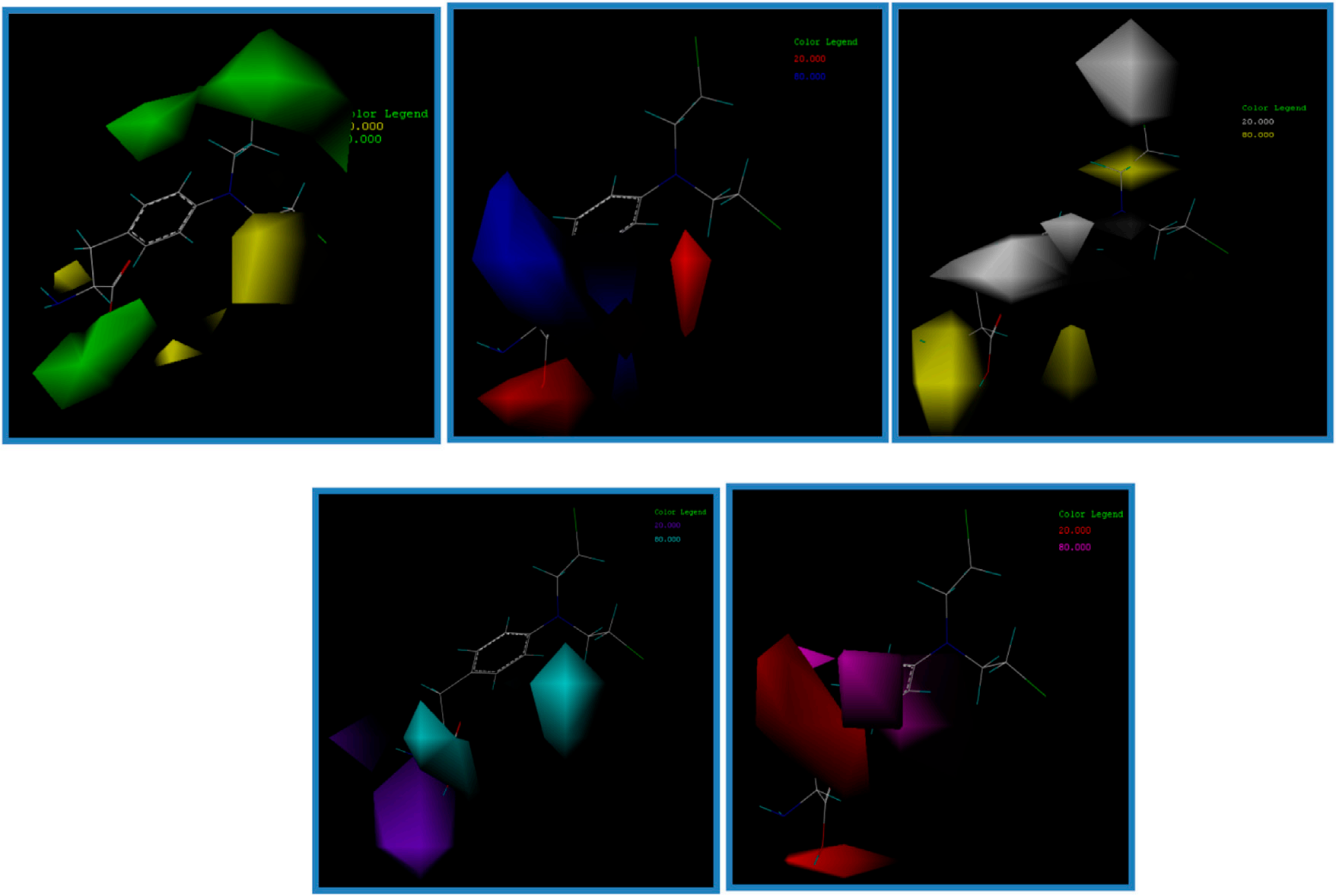
Contour map of the optimal compound I1. **(A)** In the steric field, green represents favorable and yellow represents unfavorable. **(B)** In the electrostatic field, blue represents a positive electric field and red represents a negative electric field. **(C)** In the hydrophobic field, yellow represents favorable and white represents unfavorable. **(D)** Favorable (cyan) and unfavorable (purple) hydrogen bond donor fields. **(E)** Favorable (magenta) and unfavorable (red) hydrogen bond acceptor fields.

In this experiment, contour maps of the spatial potential field, electrostatic field, hydrophobic field, hydrogen bond donor field, and hydrogen bond acceptor field of the CoMSIA model were constructed, respectively, according to compound I1 with the highest IC_50_ value (Figure 7).

The contribution value of the electrostatic field is the highest among these five contour plots, so it is necessary to focus on the construction of compounds in this field in subsequent drug design experiments.

### 3.6 The design of new compounds and the prediction of their activity

In the 2D-QSAR experimental results of nitrogen-mustard compounds, “Min electroph react index for a C atom” was found to be the most important descriptor affecting the drug activity of compounds. Therefore, the descriptor “Min electroph react index for a C atom” should be added to the idea of a new drug design so as to improve the drug activity of newly designed compounds.

Finally, 200 new nitrogen-mustard compounds were designed according to the molecular descriptor “Min electroph react index for a C atom” and the CoMSIA model contour plot (especially the electrostatic field). The IC_50_ value of these 200 new compounds was predicted using the CoMSIA model. The 10 compounds with the highest IC_50_ predictive values are listed in Table 6, among which compound I1.10 has the highest drug activity value, which may be a potential anti-osteosarcoma drug, but small molecule docking assays are still needed.

**TABLE 6:** Newly designed compounds and their predicted values.

Name	Predictive value
I1	4.569
I1.1	5.121
I1.2	5.438
I1.3	5.557
I1.4	5.669
I1.5	6.214
I1.6	6.647
I1.7	6.749
I1.8	6.789
I1.9	6.241
I1.10	6.843

Note: The structure of the compound is shown in Supplementary Material S1.

### 3.7 Molecular docking experiment

In order to prove the effectiveness of the newly designed compounds on osteosarcoma-related protein targets, a molecular docking test on small-molecule compounds and proteins was carried out using SYBYL (SYBYL-2.1.1) software package. Compound I1 and I1.10 were docked as ligands in the docking experiment. Meanwhile, as ifosfamide is the most common chemotherapy drug for osteosarcoma, it was also added as a ligand in the docking experiment.

**FIGURE 8:**
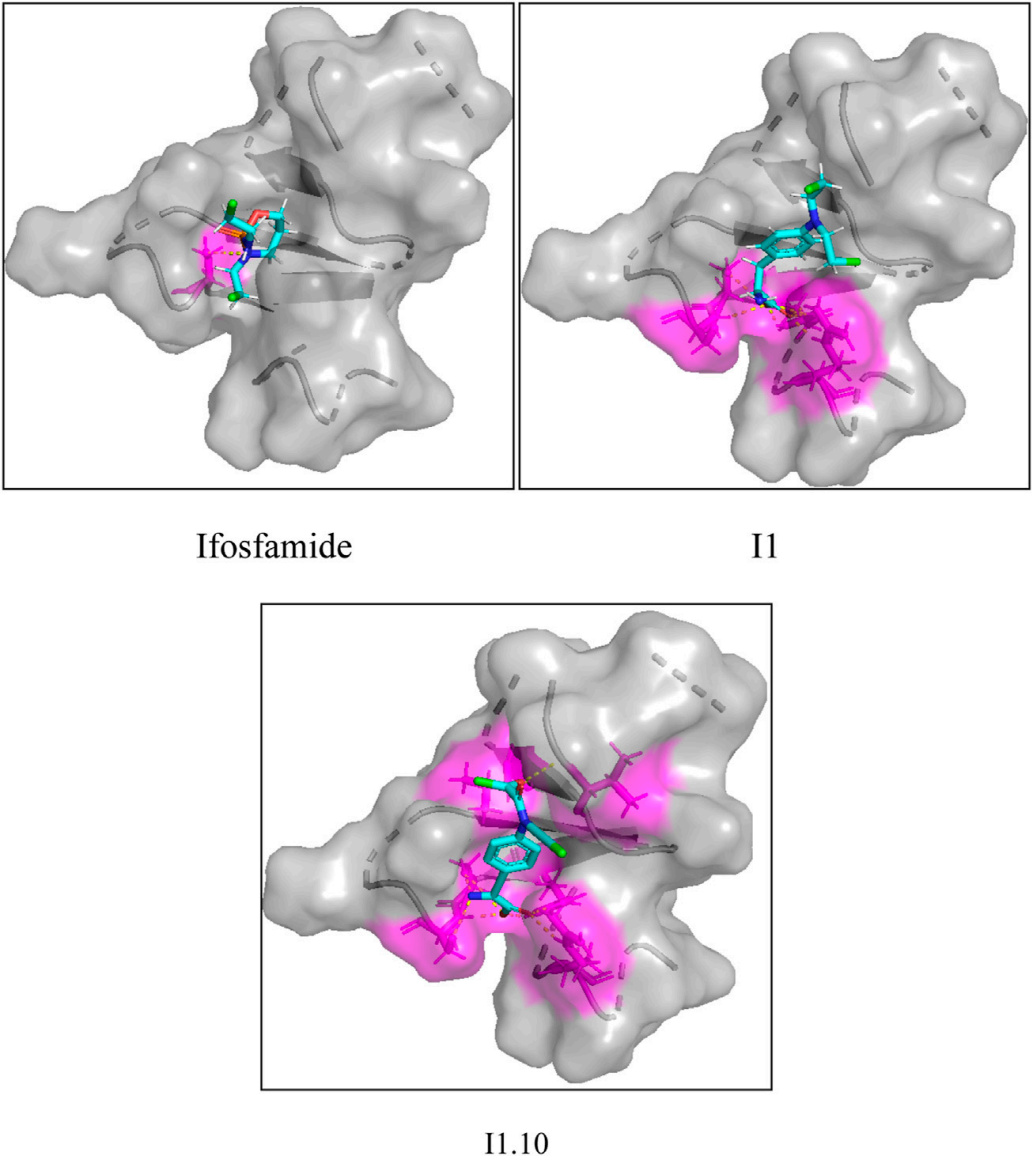
Docking experiments of ifosfamide, I1, and I1.10 with osteosarcoma targets.

In this study, a total of four transcriptional osteosarcoma-related DNA sequences were screened out, which were mTOR, OGG1, EGFR, and PDGFR-β. Four pieces of DNA produced a total of 344 protein receptors, of which only 12 had docking activity with nitrogen-mustard compounds. In this experiment, 1H90 with the best docking ability was selected as the target. The docking experiments on these three compounds are shown in Figure 8. It can be seen from the figure that compound I1.10 can form five hydrogen bonds with the protein, while the remaining two small molecules can only form one and three hydrogen bonds, while the docking fractions of ifosfamide, I1, and I1.10 are 3.495, 6.124, and 9.105, respectively; so, compound I1.10 has the best docking ability.

## 4 Discussion

First, the advantage of this experiment lies in the innovation of a QSAR research mode. In the previous QSAR experiments, QSAR models were established for tumor targets with known structures, where the compounds were designed. However, such experimental design ideas had limitations, e.g., some excellent drugs could not be extended to other tumor treatments. In order to change this limitation, in this study, the main regulatory DNA sequences of osteosarcoma were first screened and were then followed by the protein targets so as to achieve an effective inhibition of the compounds on osteosarcoma. This approach has two advantages. The first is that this approach can ensure effective docking of the compounds to target tumors, improving the ability to predict whether the compounds have an effect on the tumor. The second advantage is the improvement of the adaptability of cancer chemotherapy drugs and the increase of the diversity of cancer drugs by predicting whether there is an inhibitory effect between drugs and tumor targets.

Second, at the beginning of this experiment, we found few data on alkylated dipeptide nitrogen-mustard compounds. However, since such compounds indeed have a very large anti-tumor potential, we proved the model stability and prediction ability of the model in this experiment using the machine learning algorithm and internal validation (Kostakis and Kargas, 2021; Omoyiola, 2022). Simply put, the easiest way to conduct an internal validation of models through machine learning algorithms is to increase or decrease the data amount in training sets and test sets. Therefore, in the subsequent work, we conducted two 3D-QSAR experiments by increasing or decreasing the data amount in the training set and test set, finding that q^2^ in the two experiments was 0.552 and 0.521, respectively, which was basically similar to Q^2^ in this experiment (0.532). Therefore, the model in this experiment conformed to the basic principles of the machine learning algorithm, which also proved that it had good stability and prediction ability.

In 2D-QSAR experiments, the drug activity of compounds is mainly affected by changing the proportion of the molecular descriptors of those compounds. In a 3D-QSAR experiment on a compound, the main factor affecting its drug activity was the changes in its effective group in different force fields. In this experiment, we combined the most influential molecular descriptors in a 2D-QSAR experiment with the contour map of the 3D-QSAR model to serve as a guiding idea for designing new drugs. This drug design method has been very reliable in the research results of this experiment.

## 5 Conclusion

In this experiment, linear and non-linear 2D-QSAR models are established using the heuristic method and GEP algorithm. By comparing the two 2D-QSAR models, it was found that the non-linear model had better stability and prediction ability, but the 2D-QSAR model had a limitation, i.e., it could not be used to accurately describe the influence of the changes in the spatial structure of compounds on their anti-tumor activity. Therefore, we used the CoMSIA method to construct a 3D-QSAR model with a higher q^2^ (0.532) and r^2^ (0.997) value and a lower estimated standard error (0.016) value. By comparing the 2D-QSAR model with the 3D-QSAR model, it was found that the 3D-QSAR model could intuitively show the changes in the spatial structure and anti-tumor activity of those compounds. Finally, 200 new nitrogen compounds were constructed by combining the molecular descriptor “Min electroph react index for a C atom” of the 2D-QSAR model with the molecular force field of the 3D-QSAR model, in which compound I1.10 had the highest drug activity. However, in order to further demonstrate the effectiveness of these compounds on osteosarcoma-related receptor targets, we continued to perform small-molecule docking experiments, and the docking results of compound I1.10 were satisfactory.

## Data Availability

The original contributions presented in the study are included in the article/Supplementary Material; further inquiries can be directed to the corresponding authors.
