# Supplementary figures and images for: 3D- and 2D-QSAR models’ study and molecular docking of novel nitrogen-mustard compounds for osteosarcoma
Source: Front Mol Biosci. 2023 Mar 29;10:1164349. doi: 10.3389/fmolb.2023.1164349 (PMC10090277; doi:10.3389/fmolb.2023.1164349)

| I1 | 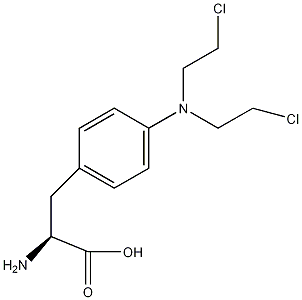 |
| --- | --- |
| I1.1 | 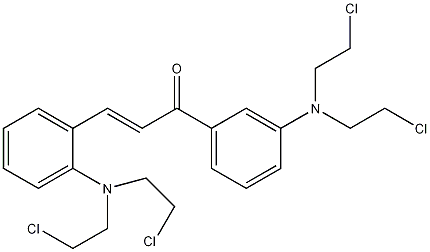 |
| I1.2 | 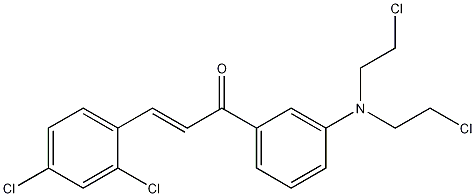 |
| I1.3 | 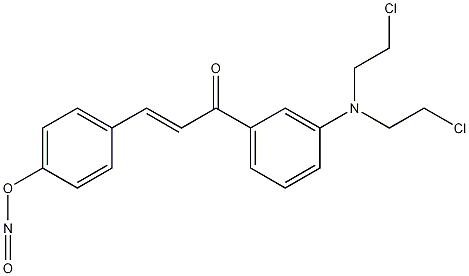 |
| I1.4 | 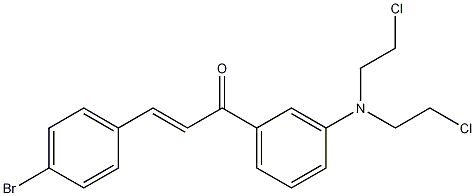 |
| I1.5 | 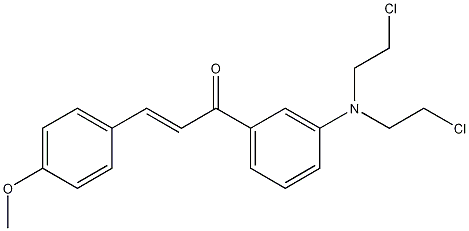 |
| I1.6 | 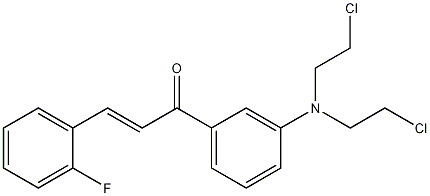 |
| I1.7 | 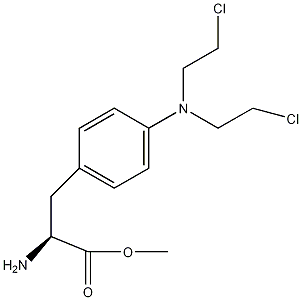 |
| I1.8 | 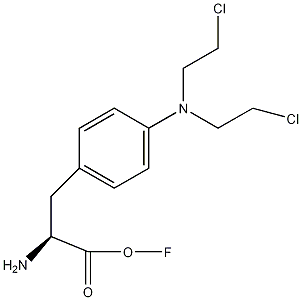 |
| I1.9 | 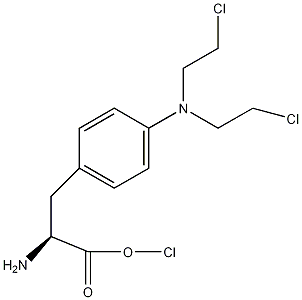 |
| I1.10 | 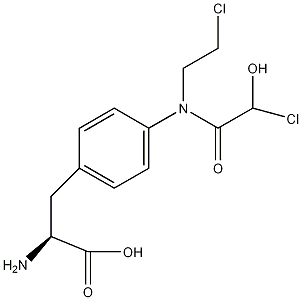 |

Supplement: Supplementary file 1 [file Table1.DOCX]

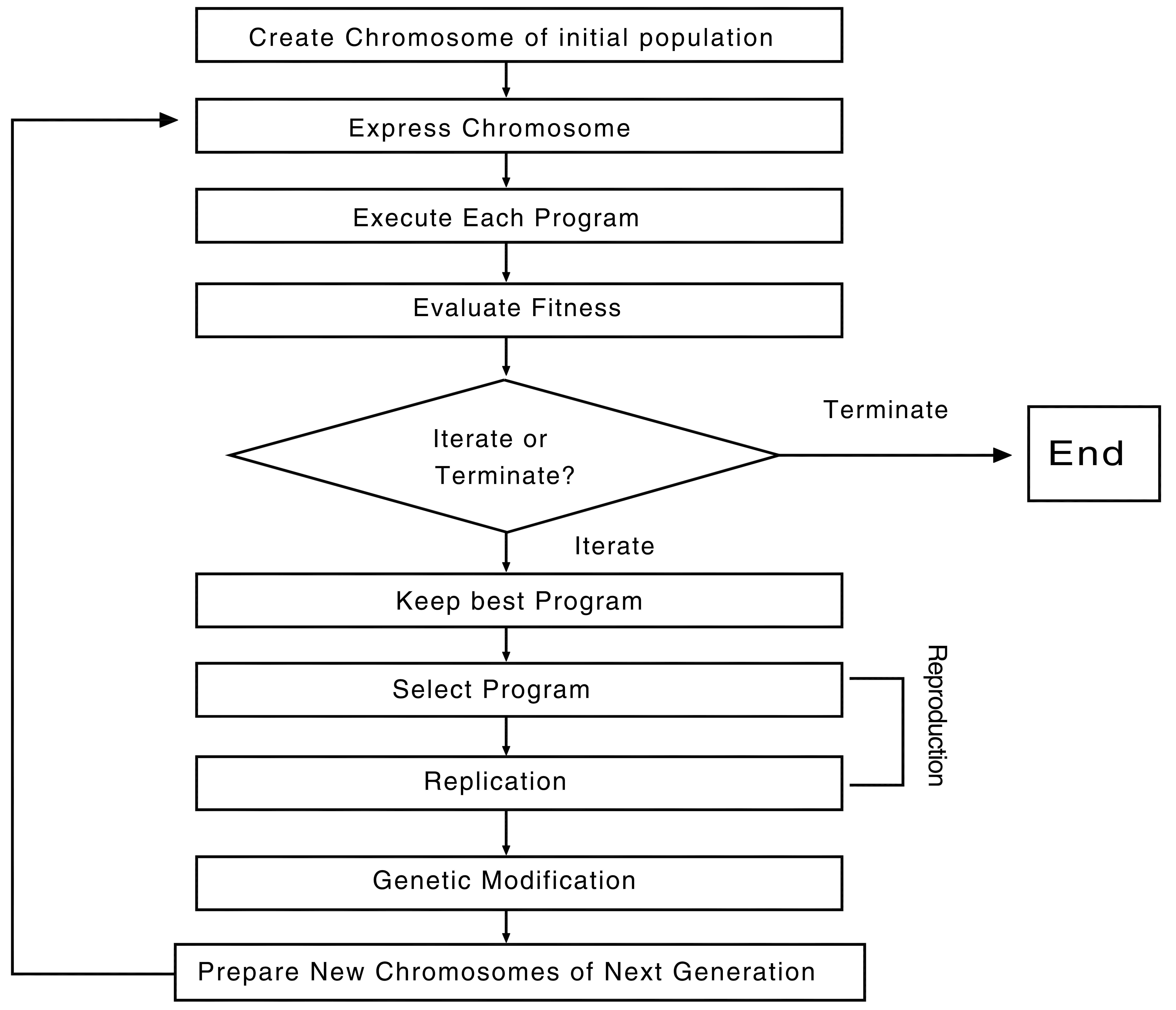

Supplement: Supplementary file 2 [file DataSheet1.ZIP › zuiz/fig1.jpeg]

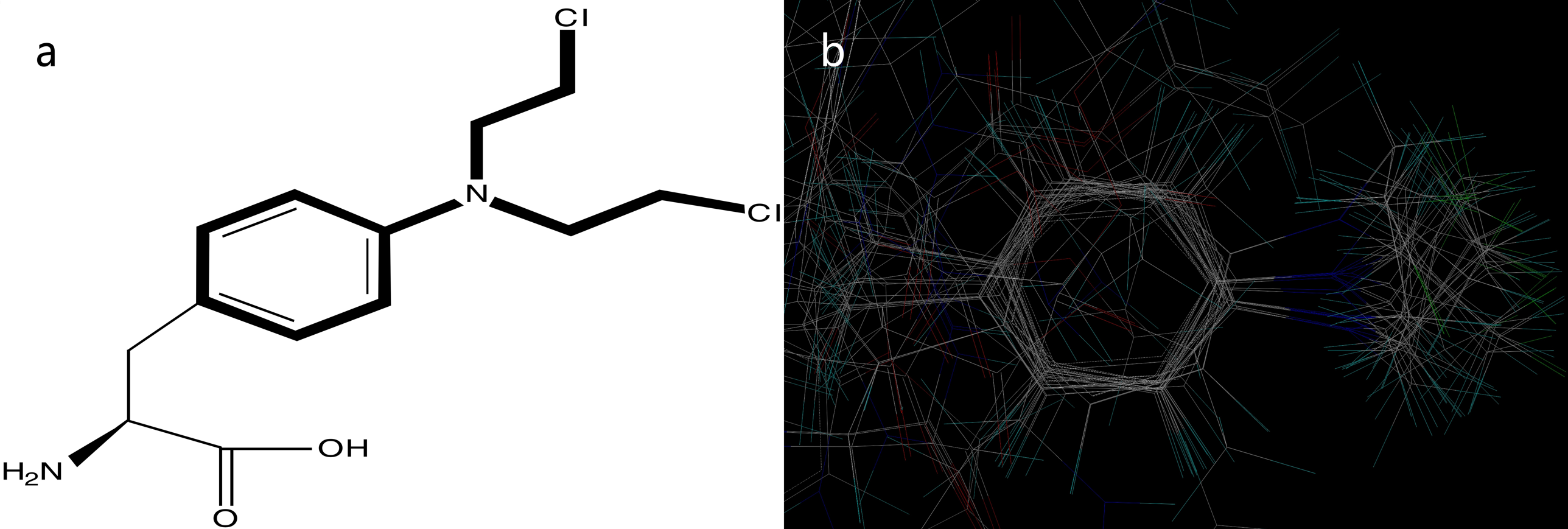

Supplement: Supplementary file 2 [file DataSheet1.ZIP › zuiz/fig2.jpeg]

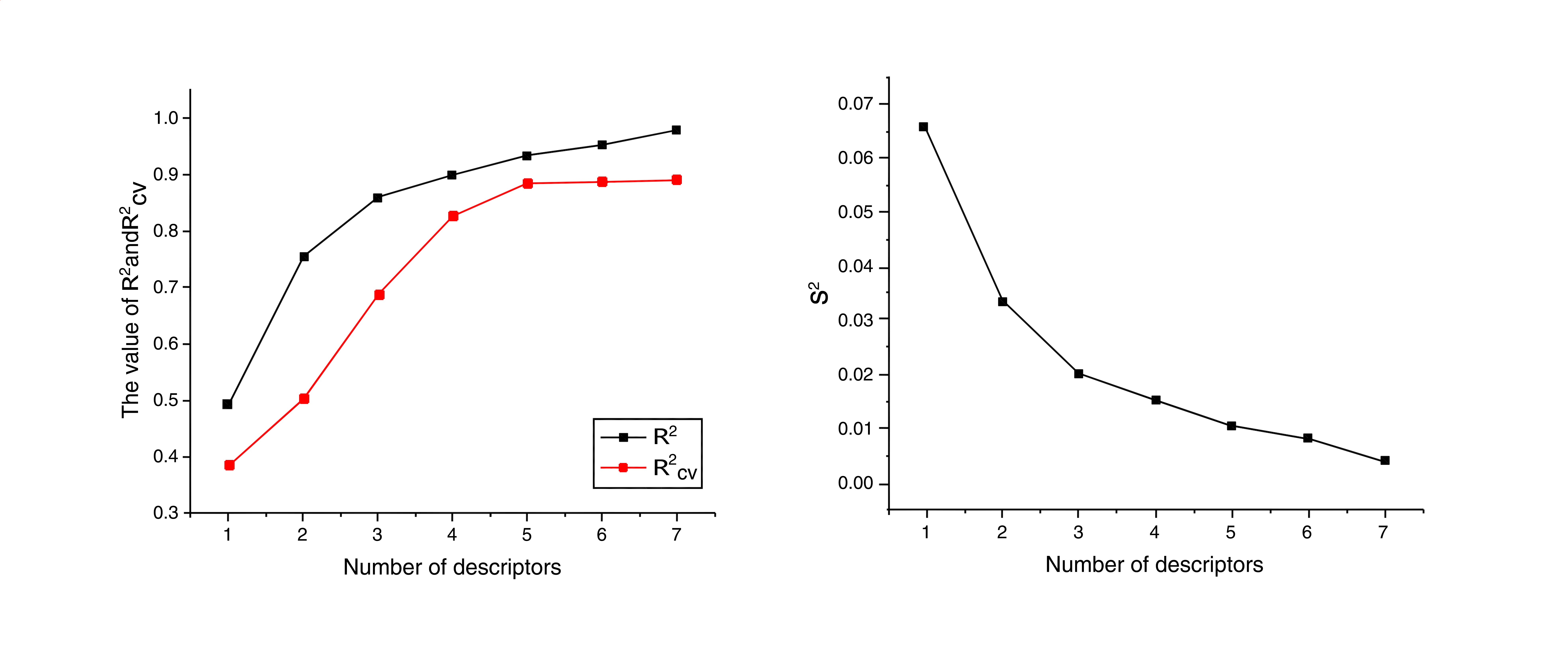

Supplement: Supplementary file 2 [file DataSheet1.ZIP › zuiz/fig3.jpeg]

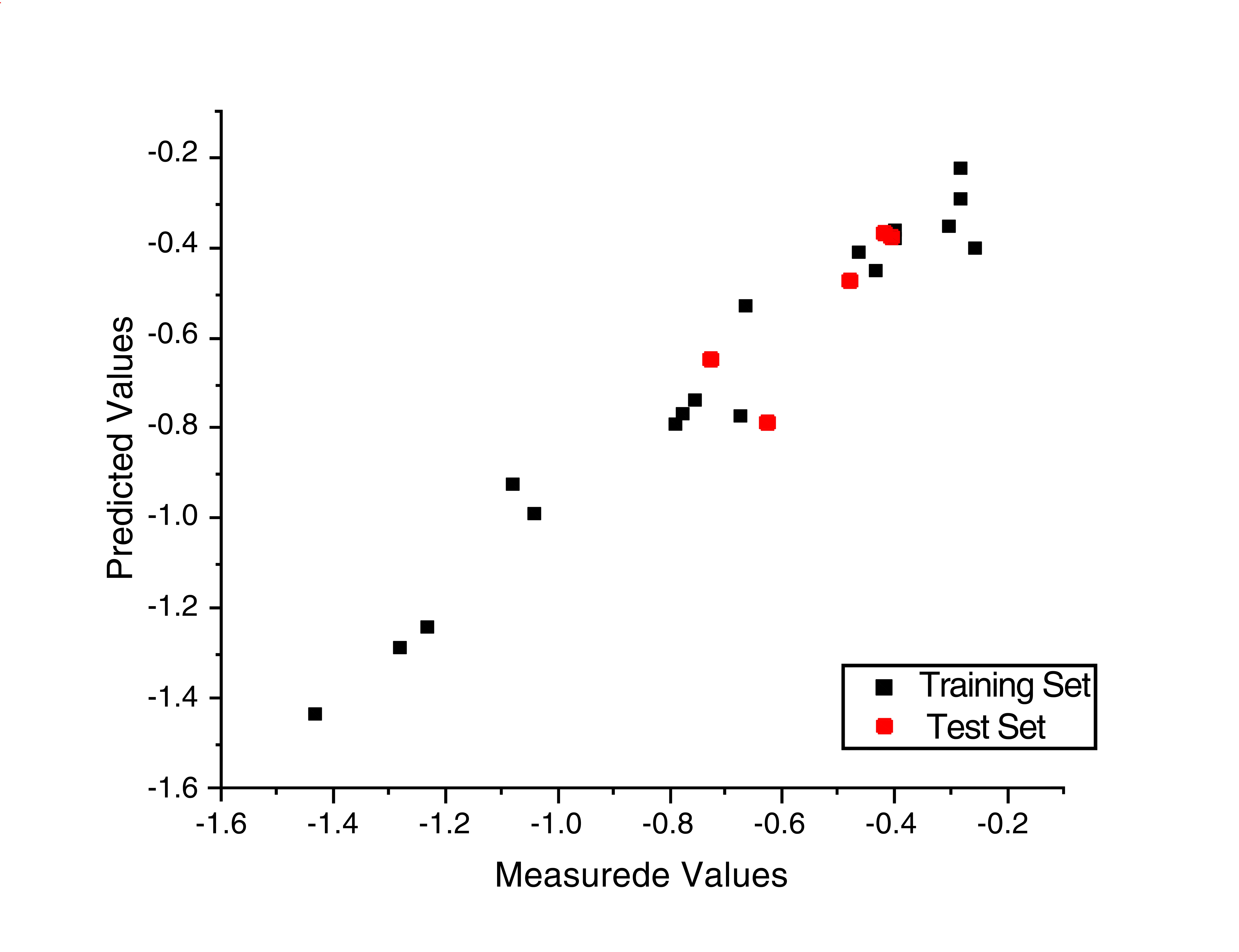

Supplement: Supplementary file 2 [file DataSheet1.ZIP › zuiz/fig4.jpeg]

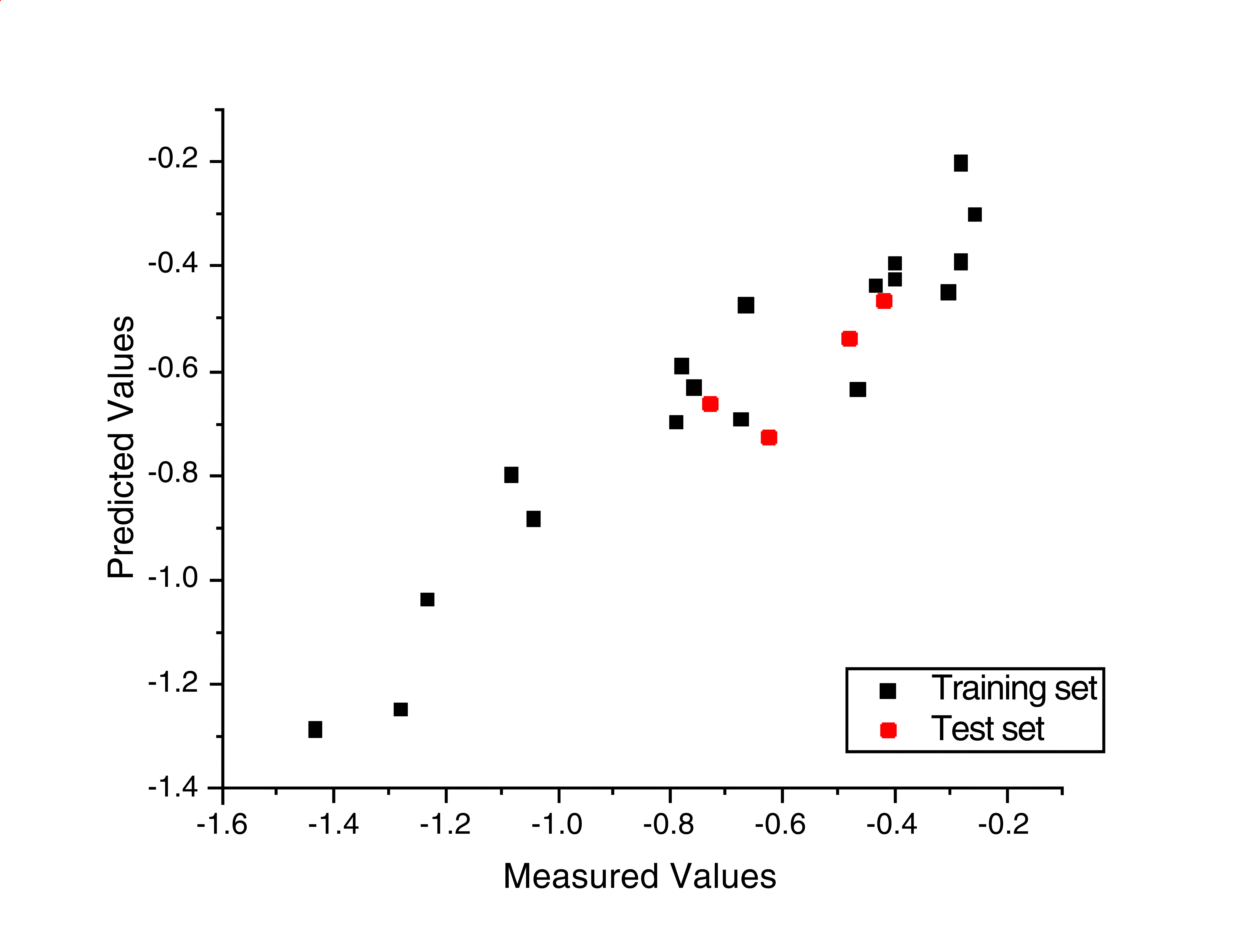

Supplement: Supplementary file 2 [file DataSheet1.ZIP › zuiz/fig5.jpeg]

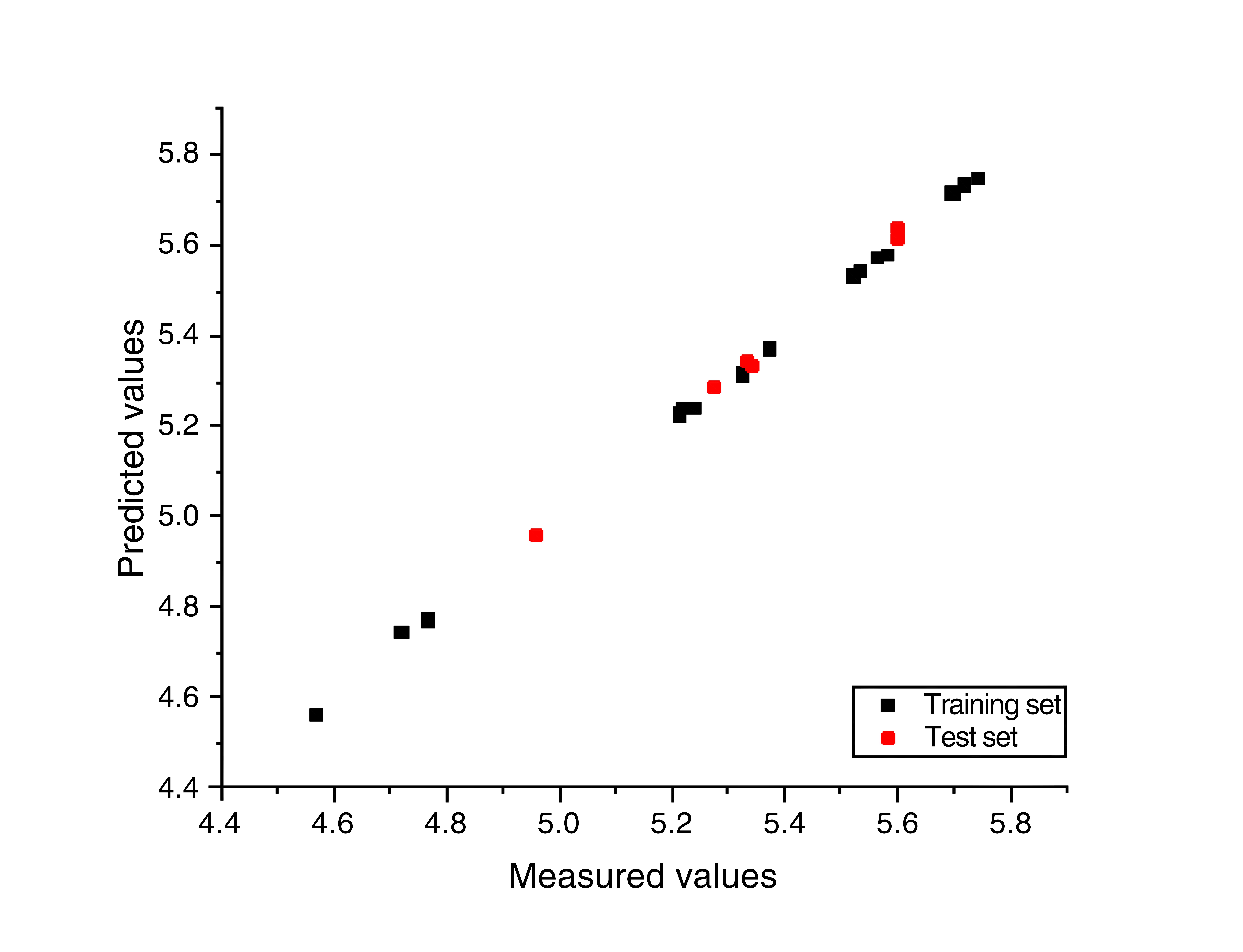

Supplement: Supplementary file 2 [file DataSheet1.ZIP › zuiz/fig6.jpeg]

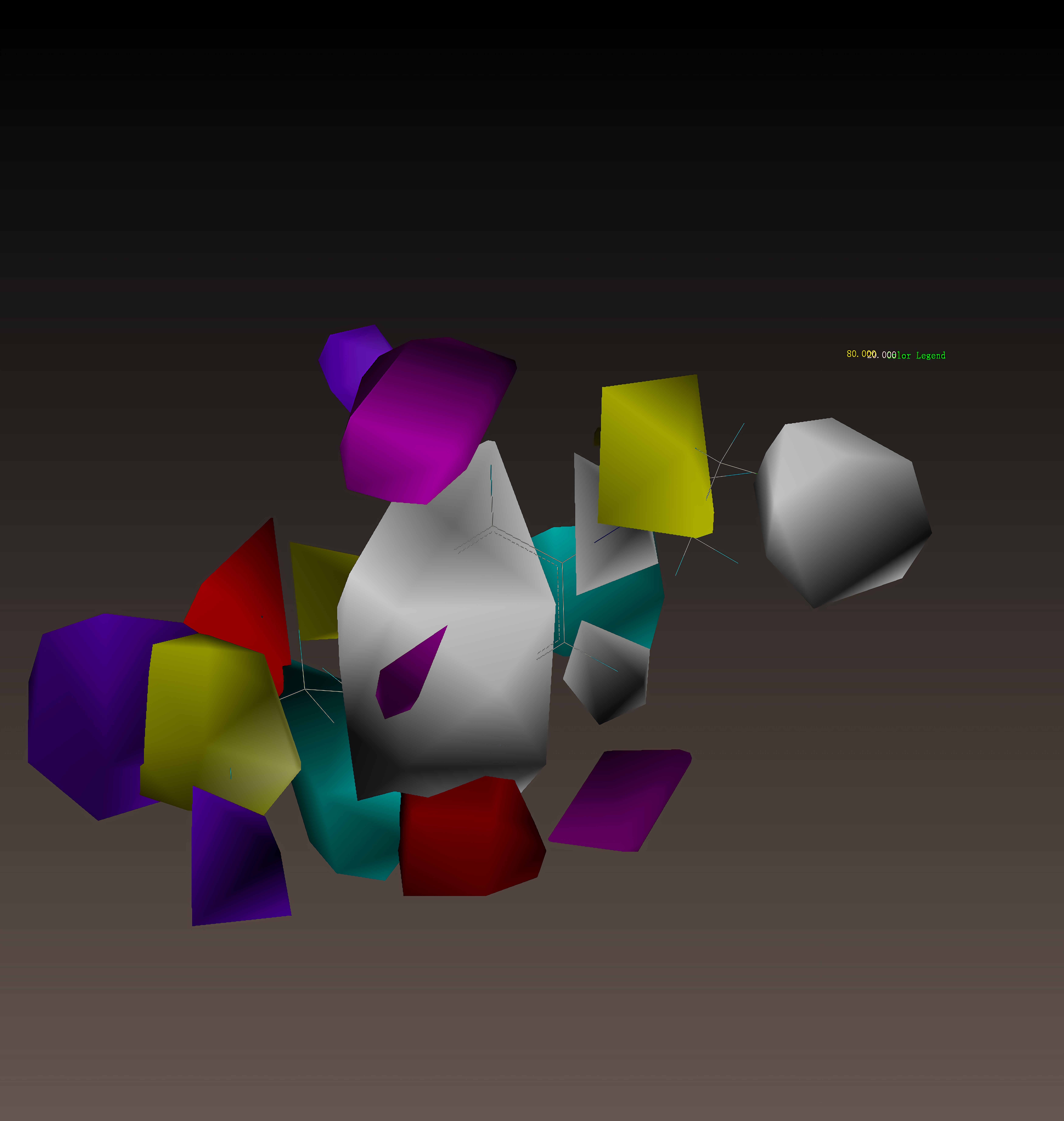

Supplement: Supplementary file 2 [file DataSheet1.ZIP › zuiz/fig7.jpeg]

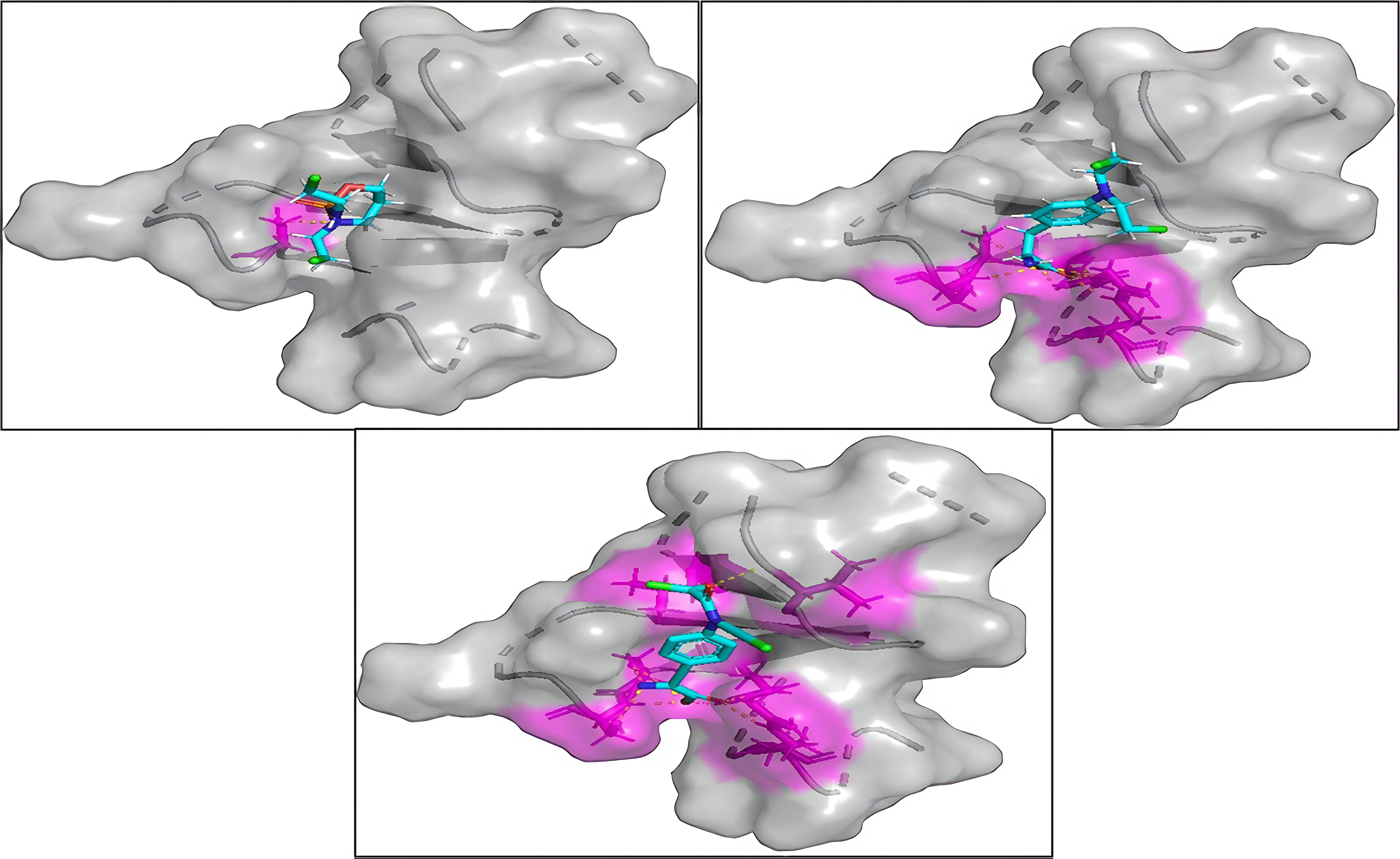

Supplement: Supplementary file 2 [file DataSheet1.ZIP › zuiz/fig8.jpeg]
